# Large effect quantitative trait loci for salicinoid phenolic glycosides in *Populus*: Implications for gene discovery

**DOI:** 10.1002/ece3.3932

**Published:** 2018-03-08

**Authors:** Scott A. Woolbright, Brian J. Rehill, Richard L. Lindroth, Stephen P. DiFazio, Gregory D. Martinsen, Matthew S. Zinkgraf, Gerard J. Allan, Paul Keim, Thomas G. Whitham

**Affiliations:** ^1^ Department of Biology University of Arkansas at Little Rock Little Rock AR USA; ^2^ Department of Chemistry US Naval Academy Annapolis MD USA; ^3^ Department of Entomology University of Wisconsin‐Madison Madison WI USA; ^4^ Department of Biology West Virginia University Morgantown WV USA; ^5^ Environmental Genetics and Genomics Laboratory (EnGGen) Department of Biological Sciences Merriam‐Powell Center for Environmental Research Northern Arizona University Flagstaff AZ USA; ^6^ Department of Biology Western Washington University Bellingham WA USA; ^7^ Department of Biological Sciences Pathogen and Microbe Institute Northern Arizona University Flagstaff AZ USA

**Keywords:** community and ecosystem genetics, defensive chemistry genes, foundation species, *Populus*, quantitative trait loci mapping, salicinoid phenolic glycoside

## Abstract

Genomic studies have been used to identify genes underlying many important plant secondary metabolic pathways. However, genes for salicinoid phenolic glycosides (SPGs)—ecologically important compounds with significant commercial, cultural, and medicinal applications—remain largely undescribed. We used a linkage map derived from a full‐sib population of hybrid cottonwoods (*Populus* spp.) to search for quantitative trait loci (QTL) for the SPGs salicortin and HCH‐salicortin. SSR markers and primer sequences were used to anchor the map to the V3.0 *P. trichocarpa* genome. We discovered 21 QTL for the two traits, including a major QTL for HCH‐salicortin (*R* ^2^ = .52) that colocated with a QTL for salicortin on chr12. Using the V3.0 *Populus* genome sequence, we identified 2,983 annotated genes and 1,480 genes of unknown function within our QTL intervals. We note ten candidate genes of interest, including a BAHD‐type acyltransferase that has been potentially linked to *Populus* SPGs. Our results complement other recent studies in *Populus* with implications for gene discovery and the evolution of defensive chemistry in a model genus. To our knowledge, this is the first study to use a full‐sib mapping population to identify QTL intervals and gene lists associated with SPGs.

## 1 INTRODUCTION

Unlike primary metabolites that govern “fundamental life processes,” secondary metabolites often mediate interactions between plants and their biotic and abiotic environments (Neuman, Kumar, & Imani, 2009). Many plant secondary metabolites have important ecological functions (Lindroth & St. Clair, 2013; Steppuhn et al., 2004) as well as economical (e.g., Pickett et al., 2016), medicinal (Devore, Kang, Breteler, & Grodstein, 2013), and ethnobotanical applications. To fully exploit the potential benefits of plant secondary metabolites, we need to know the genes that underlie the metabolic pathways governing their synthesis and regulation (Redding‐Johanson et al., 2011). Likewise, a genetic perspective of secondary metabolism is important to our understanding of how plant chemistry evolves (and coevolves) to affect the ecology and evolution of diverse organisms whose survival is directly or indirectly dependent upon the availability of suitable plant hosts (Lamit et al., 2015).

The genetic bases of metabolic pathways controlling the synthesis of many important secondary metabolites, including nicotine (Steppuhn, Gase, Krock, Halitschke, & Baldwin, 2004), various flavonoids (Saito et al., 2013), and terpenes (Redding‐Johanson et al., 2011), are well characterized. However, the pathways leading to the salicinoid phenolic glycosides (SPGs) remain almost entirely undescribed (Tsai, Harding, Tschaplinski, Lindroth, & Yuan, 2006; Tsai, Kayal, & Harding, 2006; Boeckler, Gershezon, & Unsicker, 2011; but see Chedgy, Kӧllner, & Constabel, 2015). The lack of genetic data for SPGs is conspicuous given their potential agricultural, medicinal, and ecological applications. For example, salicortin, one of the SPGs in our study, has recently been investigated for potential application toward the treatment and prevention of obesity (Lee et al., 2013), insulin resistance (Harbilas et al., 2013), and inflammation (Kwon et al., 2014). Ecologically, salicortin and other SPGs serve a variety of functions (reviewed in Tsai, Harding, et al., 2006; Tsai, Kayal, et al., 2006) that include protection from UV damage (Turtola et al., 2005; Warren, Bassman, Fellman, Mattinson, & Eigenbrode, 2003), responses to drought (Turtola et al., 2005), and defense against herbivores (e.g., Lindroth & St. Clair, 2013; Holeski et al., 2016; Tahvanainen et al., 1991). In the model tree genus *Populus*, plant chemistry has been shown to be the key bridge between host plant genetic variation and insect community organization (Bangert et al., 2006; Bernhardson et al., 2013; Martinsen et al., 1998). For example, variation in leaf salicortin and other SPGs can alter herbivory by important arthropod pests, such as gypsy moths (Couture et al., 2016; Donaldson & Lindroth, 2007), forest tent caterpillars (Hwang & Lindroth, 1997), and other herbivores (e.g., Wooley, Walker, Vernon, & Lindroth, 2008). In contrast, SPGs are sequestered by keystone, leaf‐feeding beetles of the genus *Chyrsomela* and used in their own defenses (Soetens, Pasteels, Daloze, & Kaisin, 1998; see also Kearsley & Whitham, 1992; Martinsen, Driebe, & Whitham, 1998; Waltz & Whitham, 1997). Foliar SPGs have also been shown to affect symbiotic microbes. For example, studies of aspen–gypsy moth interactions at the “foliar‐gut interface” have shown that SPGs can influence midgut community composition (Mason, Rubert‐Nason, Lindroth, & Raffa, 2015). Some midgut bacteria are able to metabolize SPGs (Mason, Lowe‐Power, Rubert‐Nason, Lindroth, & Raff, 2016) and could provide new models for investigating the evolution of symbioses as driven by plant defensive chemistry.

The examples above suggest that the ability to manipulate SPG gene expression could contribute to a better understanding of the evolution of plant chemical diversity and its consequences for other aspects of *Populus* biology, such as trade‐offs between growth and defense (Osier & Lindroth, 2006). Such an understanding is also likely to play a role in the eventual exploitation of plant genes for increased disease resistance, wood quality, herbal flavor and fragrance products, nutraceuticals, and pharmaceuticals (Tsai, Harding, et al., 2006).

The discovery of SPG genes has been hampered, in part, by their apparent absence in model herbaceous species (Tsai, Harding, et al., 2006). However, forest trees from the model genus *Populus* express a diverse array of SPGs and other secondary metabolites of interest (Boeckler et al., 2011; Chen, Liu, Tschaplinski, & Zhao, 2009; Constabel & Lindroth, 2010; Keefover‐Ring et al., 2014). *Populus* species have been studied extensively in genomic (Tuskan et al.,^2006^), metabolomic (Morreel et al., 2006; Tsai, Harding, et al., 2006; Tsai, Kayal, et al., 2006), ecological (Boeckler et al., 2011; Caseys, Stritt, Glauser, Blanchard, & Lexer, 2015; Lindroth & St. Clair, 2013), and commercial improvement (Jansson & Douglas, 2007; Taylor, 2002; Wullschleger, Jansson, & Taylor, 2002) research. The North American black cottonwood (*P. trichocarpa*) was the first forest tree to have its genome sequenced (Tuskan et al., 2006), and it and many of its congeners are valuable resources for wood fiber, carbon sequestration, and biofuels development (Taylor, 2002). Across the northern hemisphere, *Populus* species frequently act as foundation species*—*organisms that modulate and stabilize fundamental community and ecosystem processes (Ellison et al., 2005; Whitham et al., 2006) in riparian and other forest ecosystems. Ease of sexual and vegetative propagation, fast growth rate, short time to sexual maturity, and widespread interspecific hybridization predispose *Populus* to experimental manipulation, and numerous genetic mapping pedigrees have been created to study the genetic basis of many traits of commercial or ecological significance (e.g., DeWoody et al., 2013; Rae et al., 2008; Robinson et al., 2012; Woolbright et al., 2008).

Here, we used a previously published (Woolbright et al., 2008) backcrossed mapping population of naturally hybridizing cottonwoods (i.e., *P. fremontii* and *P. angustifolia*) to identify QTL associated with the SPGs salicortin and HCH‐salicortin. While the ecological effects of HCH‐salicortin have yet to be established in empirical studies using the purified compound, it differs from salicortin only in the addition of a second hydroxycyclohexane‐on‐oyl (HCH) functional group (Rehill, Clauss, Wieczorek, Whitham, & Lindroth, 2005). As this functional group confers toxicity to SPGs (Lindroth, Scriber, & Hsia, 1986), HCH‐salicortin is likely as biologically active as, or more so than salicortin and related compounds that contain the chemical moiety.

Our study was designed with three long‐term objectives in mind: First, we sought to contribute to the discovery of genes controlling an important, but largely uncharacterized metabolic pathway. Second, we aimed to complement other recent studies investigating the evolution of plant secondary chemistry in *Populus* (Caseys et al., 2015; Chedgy et al., 2015; see also Bernhardson et al., 2013). Third, we looked to establish a basis for genomic approaches to community ecology that will link changes at the DNA sequence level with variation in ecologically important chemical traits that influence other species, shape community structure, and drive ecosystem processes (Schuman, Allmann, & Baldwin, 2015). Given patterns of inheritance observed in previous studies (Bailey, Wooley, Lindroth, & Whitham, 2006; Rehill et al., 2005, 2006; Stevens & Lindroth, 2005), we made the following predictions:

1. Expression of salicortin and HCH‐salicortin should be correlated given potential precursor–product relationships or competition for shared substrates.
2. Only those progeny that received donor parent (*P. fremontii*) alleles at certain loci would express the trait because HCH‐salicortin is expressed at very low levels in some *P. angustifolia* genotypes (Holeski, Hillstrom, Whitham, & Lindroth, 2012) and is not detectable in others, including the recurrent parent genotype in our study.

## 2 MATERIALS AND METHODS

### 2.1 Pedigree and linkage map

QTL analyses were performed using a previously described mapping pedigree of hybridizing cottonwoods (Woolbright et al., 2008). Briefly, we crossed two individuals (F_1_ genotype WSU‐6 and *P angustifolia* genotype #996) from a natural population from the Weber River of northern Utah (Keim et al. 1989, Martinsen et al. 1998) to create a backcross mapping population of 246 backcross progeny. Both parental genotypes were of unknown parentage and were identified using RFLP markers. Progeny were raised under greenhouse conditions at Northern Arizona University's research greenhouse facility. Leaf samples for both genetic and chemical analyses were collected on dry ice from cuttings that were at least 2 years old and propagated in five‐gallon pots. Samples were lyophilized, ground in a Wiley mill, and stored at −20°C until use.

The mapping pedigree was used to construct a framework linkage map composed of 326 AFLP markers distributed among nineteen linkage groups (Woolbright et al., 2008). Chromosome assignments and marker order were established using MapMaker 3.0 (Lander et al., 1987) with “default linkage criteria” of LOD = 8.0 and recombination fraction (rf) = 0.37. One hundred eleven microsatellite (SSR) markers conserved across *Populus* species were also included in the complete map allowing us to anchor our linkage groups to the nineteen *Populus* chromosomes (see Tuskan et al., 2006). Thirty‐eight AFLP markers were dropped from the QTL analysis due to segregation distortion (Woolbright et al., 2008). In addition, four others were dropped due to unacceptable levels of missing data. Finally, the order of four markers was changed following reanalysis of marker data using the software package R/QTL (Arends, Prins, Jansen, & Broman, 2010; Broman & Sen, 2009, two on chromosome chr2 and two on chromosome chr18). New maps were generated using the *switch.order* function in R/QTL and the entire linkage map for all 19 chromosomes was reestimated using *est.map* function with an error.prob = 0.03 (the estimated scoring error rate for AFLP markers).

### 2.2 Chemistry data

Phenolic glycoside concentrations (salicortin and HCH‐salicortin) were determined for 172 progeny as per Lindroth, Scriber, & Hsia, 1986. Briefly, 25 mg of freeze‐dried leaf powder was placed in 1 ml of methanol at 0°C and sonicated for 30 min. The extract was analyzed by high‐performance thin‐layer chromatography using salicortin and HCH‐salicortin as standards. These compounds are the major phenolic glycosides in this system (Rehill et al., 2005); standards were purified from cottonwood leaves by liquid–liquid extraction (Lindroth et al., 1996), followed by medium‐pressure, “flash” chromatography (Still, Kahn, & Mitra, 1978). Correlation analysis (Spearman's rank) and nonlinear regression (LOESS) of the two traits were performed in R (R Core Team, 2016) and graphed using ggplot2 (Wickham, 2009). Prior to QTL analyses, salicortin data were transformed using the Box–Cox method via online software from Wessa (2016) to establish a normal distribution. All leaf salicortin and HCH‐salicortin phenotypic measurements are reported as percent dry weight (%dw).

### 2.3 QTL analyses

Salicortin QTL were identified using the MQM (multiple qtl mapping) function of R/QTL. For missing data, the most likely marker genotype was estimated via multiple imputation using the *mqmaugment* feature with the maximum number of augmented genotypes set at 1,024; a minimum probability of 0.30; and strategy = “impute.” For QTL mapping, we used the function *set.cofactors* to generate potential cofactors at 5 cM spacing across the entire linkage map. QTL were identified using the *mqmscan* function with cofactor significance of 0.002. Experiment‐wise significant thresholds (α = 0.05 and 0.10) were estimated via 1,000 permutations using the *mqmpermutation* function and used to identify significant and suggestive QTL, respectively (see Lander & Kruglyak, 1995). Chromosome‐wise significance thresholds (α = 0.10; van Ooijen, 1999) were also used to identify suggestive QTL. We further investigated significant QTL identified in the MQM analysis by fitting them to multiple QTL models (α = 0.10) using the MIM (multiple interval mapping) function of the WinQTLCartographer software (Wang, Basten, & Zeng, 2012). MIM allowed us to validate QTL peaks from the MQM analysis, refine QTL position and effect, and provided estimates of trait variance explained by each QTL (*R* ^2^).

**Figure 1:**
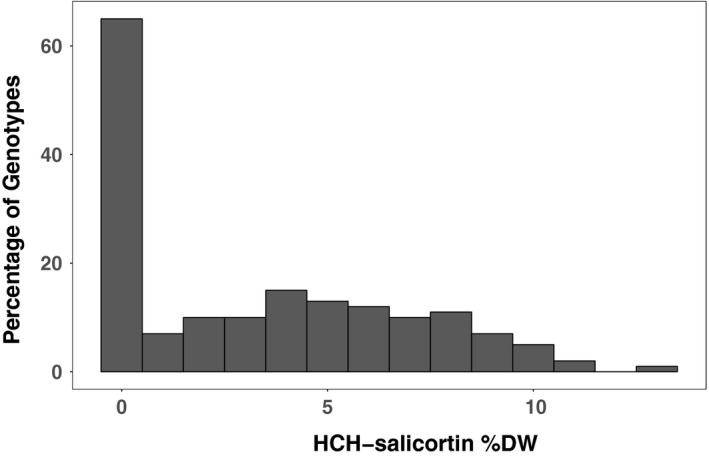
HCH‐salicortin phenotypes in the mapping population. Histogram of HCH‐salicortin measurements showing the “spike” at zero. We searched for QTL using R/qtl's “2part” model and *scanone* mapping function with “upper=FALSE” for bimodal data with a “spike” at the zero value

HCH‐salicortin is usually absent or at low concentration in most *P. angustifolia* genotypes, including our recurrent parent (genet #996), but is present in nearly all *P. fremontii* and F_1_ hybrids studied to date (Holeski et al., 2012; Rehill et al., 2005, 2006). As such, it is possible that differences among *Populus* species largely stem from simple (i.e., Mendelian) modes of inheritance. For example, a backcross toward *P. angustifolia* is predicted to result in a bimodal, “spiked” distribution where approximately one‐half of the progeny (those receiving the recurrent parental allele) will fail to express the trait. Our data followed such a distribution (Figure 1) and were not suitable for traditional mapping analyses that require normal distributions. Thus, we used R/qtl's *scanone* function with the “2part” nonparametric model for bimodal traits and “upper=FALSE” (indicating a “spike” at the zero value). The experiment‐wise LOD threshold for HCH‐salicortin was determined by 1,000 permutations. Although HCH‐salicortin data were indeed bimodal, we obtained rough estimates of QTL effect and percent trait variance explained by the major HCH‐salicortin QTL using the composite interval mapping (CIM) function of the WinQTLCartographer software.

### 2.4 Candidate genes

Using linear interpolation to align our map with the *Populus trichocarpa* genome sequence (Tuskan et al., 2006), we created lists of candidate genes near each QTL peak. We aligned QTL intervals with the genome sequence (V.3.0) using BLASTN (Altschul, Gish, Miller, Myers, & Lipman, 1990) to determine the physical locations of syntenic SSR markers. Specific criteria for accepting SSR positions were hits with a minimum length of seventeen base pairs, seventy percent (0.70) minimum identity, and agreement with expected SSR product size. Using that information, we aligned the physical positions of SSR markers with their positions in the framework map (Woolbright et al., 2008), and used regression to estimate the physical locations of each QTL interval. When QTL intervals included chromosome ends, we used either zero or total chromosome lengths (cm vs. bp) for the relevant flanking positions.

Gene lists were created using the *Populus trichocarpa* V3.0 sequence and the Biomart function on the Phytozome v12.1 database (Goodstein et al., 2012). We compiled lists from the 2.6‐Mb intervals centered on each QTL peak which corresponded to the average ±1‐LOD “drop” for the significant QTL (approximately 15 cM). By definition, LOD scores for suggestive QTL are relatively low and can result in LOD drops that are prohibitively large. Using the *P. trichocarpa* genome to create gene lists for a map based on *P. fremontii* (our donor parent) presented challenges that are discussed further below.

## 3 RESULTS

### 3.1 Salicortin

**Figure 2:**
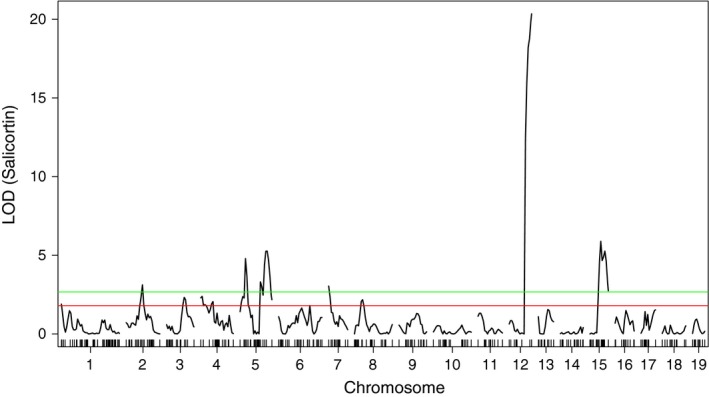
LOD scores for salicortin at all nineteen chromosomes. Horizontal lines represent the experiment‐wise (green, α = 0.05) and chromosome‐wise suggestive (red, α = 0.10) QTL thresholds

**Figure 3:**
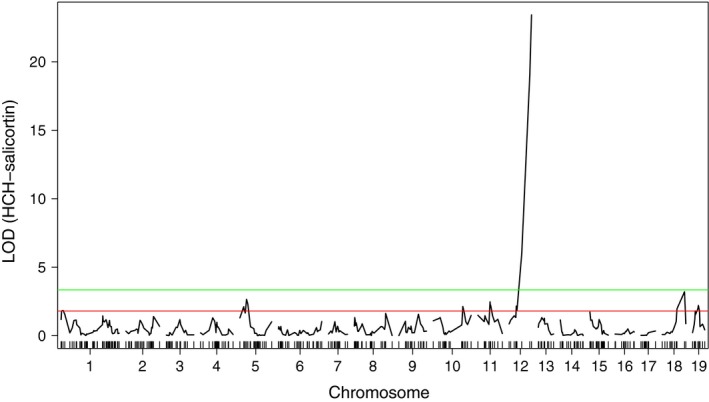
LOD scores for HCH‐salicortin at all nineteen chromosomes. Horizontal lines represent the experiment‐wise (green, α = 0.05) and chromosome‐wise suggestive (red, α = 0.10) QTL thresholds

Leaf salicortin levels in the experimental backcrosses differed by 14‐fold, ranging from 1.9 to 26.8 percent dry weight (% dw), with an average of 14.1% dw (σ = 4.94). After Box–Cox transformation to establish a normal distribution, multiple QTL mapping (MQM) revealed ten significant (LOD ≥ 2.67, α = 0.05) peaks for salicortin (Figure 2), and one (LOD ≥ 3.34) for HCH‐salicortin (Figure 3). However, we chose to treat these as seven QTL because the +/1 1.3 Mb flanking sequences used to create our 2.6‐Mb intervals overlapped for several peaks (see Table 1). In addition, we identified two suggestive QTL for salicortin (LOD ≥ 2.40) using MQM mapping (α = 0.10) and six using van Ooijen's (1999) chromosome‐wise method (LOD ≥ 1.8, α = 0.10). An additional six QTL for HCH‐salicortin were also identified using the chromosome‐wise threshold (LOD ≥ 1.8). QTL were distributed across 14 chromosomes. Data for significant peaks are summarized in Table 1. Five of the eleven significant QTL peaks were retained by the MIM model and are summarized in Table 2. For those five, *R* ^2^ values ranged from .03 to .13, and QTL effect varied where substitution of a *P. fremontii* allele resulted in a decrease in salicortin for three QTL and an increase for the remaining two. When both significant and suggestive QTL were included, substitution of *P. fremontii* alleles varied in effect, with nine QTL showing an increase in the trait, and nine showing a decrease. QTL effects ranged from 1.9% dw to –2.34% dw.

**Table 1:** Results of MQM analyses. Peaks were identified using R/qtl's MQM (salicortin) and “2‐part” (HCH‐salicotin) models (α = 0.05). LOD thresholds were ≥2.67 for salicortin and ≥3.34 for HCH‐salicortin. QTLs are named for the chromosome on which they occur and the order in which they were discovered. For example, QTL Sal_4.2 represents the second QTL described for chr04. Pos(cM) = position on the linkage map. Pos (Mb) = position in the genome. LOD is the maximum LOD score at the QTL peak. Column labeled “a” indicates the phenotypic effect (increase or decrease of the trait value) when the donor parent (*P. fremontii*) allele is substituted for the recurrent parent (*P. angustifolia*) allele at each QTL. QTL from Caseys et al. (2015) and the SPGs they correspond to were included when they occurred on the same chromosomes. Associated SSR is the marker nearest the QTL described by Caseys et al. (2015) and pos(Mb) is that marker's position in the v3.0 genome. Periods (“.”) denote data on associated marker or QTL

QTL	pos (cM)	pos (Mb)	LOD	a	*P. tremula* and *P. alba QTL* (Caseys et al., 2015)	Associated SSR	Pos (Mb)
Sal_2.1	62.50	8.77	3.14	+	.	.	.
Sal_5.1	22.63	3.77	4.82	−	***.***	.	.
Sal_5.2	79.79	15.81	3.33	+	.	.	.
Sal_5.3	100.60	20.58	5.41	+	Tremuloidin, tremulacin isomer	W15	25.78
Sal_7.1	0	0	3.04	−	Salicortin Isomer 2	O312	3.35
Sal_12.1	74.00	13.08	18.86	−	Acetyl‐salicortin isomer 1, Isomer 2	G1186	12.95
Sal_12.2	77	14.00	19.89	−	Acetyl‐salicortin isomer 1, Isomer 2	G1186	12.95
HCH_12.1	82.66	14.94	25.53	+	Acetyl‐salicortin isomer 1, Isomer 2	G1186	12.95
Sal_15.1	46.80	9.32	6.17	+	***.***	.	.
Sal_15.2	52.57	10.43	5.99	+	.	.	.
Sal_15.3	58.49	11.58	5.33	+	.	.	.

**Table 2:** QTL retained in the MIM Model. MQM QTL peaks that were also supported by MIM model‐fitting (α = .10). Columns are as in Table 1, with the addition of a column for the *R* ^2^ value for each QTL as estimated by MIM mapping

QTL	pos (cM)	LOD	a	*R* ^2^	*P. tremula* and *P. alba* QTL (Caseys et al., 2015)	Associated SSR
Sal_2.1	53.0	3.23	1.96	.09	.	.
Sal_5.3	96.4	2.68	1.90	.06	Tremuloidin, tremulacin isomer	W15
Sal_7.1	0.0	2.19	1.81	.06	Salicortin Isomer 2	O312
Sal_12.1	58.9	6.07	2.35	.13	Acetyl‐salicortin isomer 1, Isomer 2	G1186
Sal_15.1	46.8	1.32	1.57	.043	***.***	.

Suggestive QTL are summarized in Table 3 (only the sign of the QTL effect is included). Estimates of effect and *R* ^2^ were not calculated for suggestive QTL given inherent difficulty with fitting large numbers of QTL to MIM models and the fact that estimates based on suggestive QTL are less likely to be accurate (see Beavis, 1994). Tables 1 and 3 also include SSR markers that are shared with the association study by Caseys et al. (2015).

**Table 3:** Suggestive QTL from the MQM analyses and chromosome‐wise thresholds. Data are arranged as per Table 1, but for suggestive rather than significant peaks

QTL	pos (cM)	Pos (Mb)	LOD	a	*P. tremula* and *P. alba QTL* (Caseys et al., 2015)	Associated SSR	Pos (Mb)
Sal_1.1	0.00	0.00	1.90	+	.		.
HCH_1.1	10.40	2	1.87	+	.		.
Sal_3.1	69.50	16.14	2.39	−	Acetyl‐salicortin, isomer 1, isomer 2; tremuloidin; tremuloidin; tremulacin isomer	G1869	15.28
Sal_4.1	6.84	1.62	2.45	−	.		.
Sal_4.2	44.91	6.54	2.06	−	Salicortin isomer 3	O127	6.64
Sal_5.4	12.02	2.24	2.40	−	.		.
HCH_5.1	16.20	2.84	2.31	−	.		.
HCH_5.2	26.56	4.34	2.65	−	.		.
Sal_6.1	112.62	23.10	1.93	−	HCH‐tremulacin	O369	23.66
Sal_8.1	31.92	3.70	2.34	+	HCH‐Salicortin	O374	6.69
HCH_11.1	56.00	10.83	2.44	+	Tremulacin	G1037	6.99
Sal_17.1	20.52	3.11	1.97	+	.		.
HCH 18.1	76.62	15.03	2.91	−			
HCH_19.1	45.10	10.4	2.22	+	.		.

### 3.2 HCH‐salicortin

HCH‐salicortin ranged from 0.0 to 13.3% dw in the experimental backcross, with an average of 3.4% dw and a standard deviation of 3.5. Supporting our second prediction, we identified a major QTL on chromosome chr12 (LOD = 23.4) that colocated with a salicortin QTL and that explained at least 52% of the variance. In addition, we identified six suggestive QTL (LOD ≥ 1.8, Table 3). Substitution of *P. fremontii* alleles varied in the direction of effect, with an average increase in HCH‐salicortin for the major QTL on chr12. For the six suggestive QTL, three resulted in an increase in the trait, and three resulted in a decrease.

**Figure 4:**
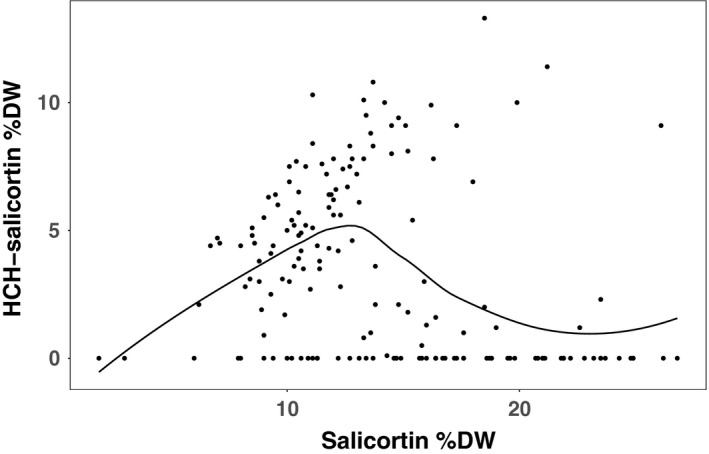
Salicortin and HCH‐salicortin correlation. A LOESS line fitted to the raw salicortin and HCH‐salicortin measurements across all individuals illustrates the complex relationship between the two traits

There was no simple linear relationship between salicortin and HCH‐salicortin as illustrated in Figure 4. While a Spearman's rank test showed that salicortin and HCH‐salicortin were negatively correlated (*r*_s_ = −0.2903, *p* < .0001), a LOESS line (nonlinear regression) fitted to the data illustrates complexity in the relationship where the two traits are positively correlated from low‐ to midrange values, but are negatively correlated when salicortin levels are highest. However, note that several individuals expressed both traits at high values.

### 3.3 Gene lists

The total number of genes and transcripts in our 2.6 Mb QTL intervals was 4,463, representing approximately 10.8% of the total genes (41, 335) for the *Populus* V3.0 sequence (see Sundell et al., 2015). Our lists include genes models that lack general identifiers or descriptions. When relevant, the positions of SSR markers used in the study by Caseys et al. (2015) were also included. Table S1 provides total chromosome‐wise gene lists and includes locations of our QTL peaks and SSRs from Caseys et al. (2015) as red text.

We identified eight genes that are associated with secondary metabolites (primarily hydroxycinnamate precursors and derivatives) and thought to play roles in predicted steps in the SPG pathway (see Tsai et al., 2006a,b) or similar metabolic pathways (see Bonpart et al., 2015). Candidate genes are summarized on Table S2 and include two BAHD‐type acyltransferases, eight *cinnamoyl‐coA reductases*, and *Shikimate dehydrogenase*. Supplemental tables are available from the Dryad Digital Repository: https://doi.org/10.5061/dryad.8h67t.

## 4 DISCUSSION

In this study, we identify several QTL associated with the SPGs salicortin and HCH‐salicortin. The most noteworthy QTL occur at the distal end of chr12 and overlap for the two traits. The major QTL for HCH‐salicortin on chr12 represent a likely Mendelian locus, and, as such, it could prove to be an important starting point for identifying undescribed SPG genes. Our results also complement other recent studies focusing on association mapping of metabolomic data (Caseys et al., 2015) including gene expression and function of candidate genes associated with SPG synthesis (Chedgy et al., 2015; see also Tsai et al., 2016a,b). Data from our study create foundations for verifying the results of future investigations of the genetic basis of SPG synthesis and the ultimate discovery of SPG genes.

### 4.1 Identification of suggestive versus significant QTL

Choice of mapping strategy can influence the identification of QTL, especially where small populations are involved. We chose to search for suggestive QTL using relaxed LOD thresholds (α = 0.10) for both the MQM mapping (Arends et al., 2010; Broman & Sen, 2009) and chromosome‐wise threshold (van Ooijen, 1999) methods in order to increase the number of QTL identified. That choice, as well as the failure to retain all loci in the MIM model, resulted in 14 of 21 QTL being categorized as “suggestive” rather than “significant” (see Lander & Kruglyak, 1987). In the past, concern that erroneous QTL (i.e., type I errors) would lead to “bloated literature” has led to relatively conservative suggestions for setting QTL thresholds (see Lander & Kruglyak, 1994). However, given the exploratory nature of most QTL studies, Beavis (1998) noted that, in the long run, type II errors (missing QTL) might be costlier than type I errors (false QTL; see also Holland, 2005), despite his own work demonstrating the increased risk of type I errors when dealing with small populations (i.e., the so‐called Beavis Effect). Likewise, Lander & Kruglyak (1994) did not completely dismiss suggestive QTL, but called for caution when reporting them. Today, when whole genomes and massively parallel sequencing make it possible to assess entire transcriptomes (e.g., RNA‐seq), virtually any QTL might prove useful if it helps to validate gene expression data. In keeping with the standards set by Lander and Kruglyak (1995), we acknowledge the “tantalizing but unproven” nature of our suggestive QTL, with an eye toward ongoing and future genomic and transcriptomic research in *Populus*. Thus, we chose to include *all* intervals and their corresponding gene lists in this article.

### 4.2 Trait variation and correlation between salicortin and HCH‐salicortin

For overlapping QTL intervals, *P. fremontii* alleles resulted in decreased values for both traits for the QTL on chr05. In contrast, substitution of *P. fremontii* alleles at our largest QTL (sal 12 and HCHsal 12) increased HCH‐salicortin but decreased salicortin. While this could be explained by precursor–product or competitive relationships, the lack of a linear correlation between the two traits (Figure 4) prevents us from accepting our first prediction that the two traits would be correlated in a manner explicable by our QTL data (e.g., by the conversion of salicortin to HCH‐salicortin as governed by a QTL such as occurs on chr12). Instead, it appears that the relationship between the two traits is complex and understanding their relative fluxes requires a broader metabolomic perspective that was outside the scope of our study (but see Tsai et al., 2006a; Casey's et al., 2015) . To date, the SPG pathway remains poorly described, underscoring the need for a genomic basis of SPG synthesis and flux.

Variation in the sign of the QTL effects for each trait could result from species‐level differences (e.g., preferred pathways, substrate specificity, allelic, or isozyme efficiency) or from similar allelic variation within the wild (outcrossed) parental genotypes. On average, salicortin levels are higher in *P. angustifolia* than *P. fremontii*; however, they vary considerably within and among species and hybrids (Rehill et al., 2005, 2006). Despite the overlap in salicortin levels, we were able to identify QTL potentially reflecting allelic or isozyme differences between hybridizing *Populus* species. In contrast, the fact that *P. angustofolia* does not produce HCH‐salicortin allowed us to identify six potential QTL for HCH‐salicortin, including a major locus on chr12 where substitution of a *P. fremontii* allele appears to be required for expression of the trait (thus supporting Prediction 2, above). The concentration of leaf HCH‐salicortin differs significantly among *Populus* generic sections, and our QTL data complement the only other published study that identifies QTL intervals associated with SPGs, including salicortin and HCH‐salicortin (Caseys et al., 2015). Likewise, our HCH‐salicortin QTL data support one other study that investigated specific candidate genes that could be directly involved with SPG synthesis (Chedgy et al., 2015; discussed below).

### 4.3 Candidate genes

Our study was intended to generate preliminary data on regions of the *Populus* genome associated with SPG synthesis. Given the length of our QTL intervals, the candidate genes discussed here are informed speculation at best, and so conclusions should be treated with caution. However, our choice to further discuss specific genes is supported by previous studies. For example, starting with the strongest QTL on chr12, we searched for candidate genes that were orthologous with other plant genes controlling similar metabolic pathways, especially those that have been tentatively linked to proposed steps within the *Populus* SPG pathway (Chedgy et al., 2015; Tsai, Harding, et al., 2006; Tsai, Kayal, et al., 2006). Our search revealed two hydroxycinnamoyl/benzoyltransferases that belong to the BAHD‐type acyltransferase family of genes Potri.001G042900 and Potri.012G144500. The first (on chr01) encodes HCT6 which is associated with lignin biosynthesis in vascular tissues (Touominen, Johnson, & Tsai, 2011; Tsai, Harding, et al., 2006; Tsai, Kayal, et al., 2006; Wegrzyn et al., 2010) and has not been shown to affect SPGs. In contrast, the function of Potri12G144500, which occurs within the interval for our strongest QTL (on chr12), remains undescribed. However, others have noted similar N‐hydoxycinnamoly/benzoyltransferases that have substrates or products that are also thought to play roles in SPG expression via control over the synthesis of benzyl benzoate and salicyl benzoate—possible intermediates in the SPG pathway (Chedgy et al., 2015; see also Bapst, Harding, & Tsai, 2010). Interestingly, Potri.012G144500 is one of the final fourteen transcripts identified for that end of chromosome chr12 under the current (V3.0) annotated genome sequence. While MQM mapping resulted in slight map expansion due to the introduction of pseudomarkers, CIM of the original, nonimputed maps placed QTL peaks for salicortin and HCH‐salicortin at terminus of the linkage map for chromosome chr12—which provides intriguing, albeit circumstantial support for candidate gene status. These findings are important because Chedgy et al. (2015) showed that enzyme activity of similar genes (e.g., *PtACT49* on chromosome chr19) is consistent with hypothesized steps in the SPG pathway (see Tsai, Harding, et al., 2006; Tsai, Kayal, et al., 2006).

Several other candidate genes identified in our study are linked to pathways that might be coregulated or otherwise linked with SPG synthesis. Examples include *shikimate dehydrogenase* (chr05) which catalyzes the conversion of 3‐dehydroshikimate to shikimate—a key entry point to the shikimic acid pathway (aka phenylpropanoid pathway). That pathway eventually leads to production of phenylalanine, which is deaminated to produce cinnamic acid—very likely an initial precursor for PGs (Tsai, Kayal, et al., 2006). Eight *cinnamoyl CoA‐reductase* (*CCR*) genes were found on (chr01). Cinnamic acid is thought to be one of the likely starting points of the SPG pathway (Tsai, Harding, et al., 2006; Tsai, Kayal, et al., 2006). Cinnamic acid leads to production of benzoyl‐CoA, which can be catalyzed by *CCR* to produce benzaldehyde. Benzaldehyde can then be metabolized to salicylaldehyde which is also one of the several precursor compounds (another being salicylic acid) that could lead to production of SPGs. While these compounds and genes create potential starting points for understanding SPG metabolism, it is important to emphasize that the main pathway(s) leading to remains unresolved.

### 4.4 Alignment with the *P. trichocarpa* genome

Creating candidate gene lists was challenging given the lack of a genome sequence for our parent species. This was particularly true for *P. fremontii*, our donor parent and source of the marker alleles used to create our map. While *P. angustifolia* is from the same generic section as *P. trichocarpa* (sect. Tacamahaca), *P. fremontii* is within a different section (sect. Aigeiros). However, previous studies have shown high levels of synteny among *Populus* species from different generic sections, as well as a high transferability of SSR markers (Berlin et al., 2010). Still, small‐scale differences between our map and *P. trichocarpa* have been noted (see Woolbright et al., 2008) and could be the product of real differences among species/sections or the result of typical mapping errors—especially those associated with relatively small sample sizes. Thus, in order to choose the size of the interval used to search for candidate genes, we had to balance the need for concise gene lists with the possibility that our QTL intervals did not align precisely with the reference genome. We created gene lists from physical intervals of 2.6 Mb, which corresponded to the average ±1‐LOD drop around our significant QTL peaks. In addition, two of our intervals contained SSR markers shared with the recent SPG association mapping study of Caseys et al. (2015). This supports the notion that these loci could be useful starting points for future genetic and genomic studies of SPG synthesis, especially those employing expression‐based approaches such as eQTL mapping, RNA‐seq and knockdown/out/up studies.

### 4.5 Ecological significance

Poplars are foundation trees, and population‐level variation in leaf chemistry is one of the strongest drivers of associated arthropod community organization (Bangert et al., 2006; Bernhardson et al., 2013; Martinsen et al., 2006). Variation in the distribution and abundance of foundation arthropods can, in turn, affect multitrophic‐level interactions with consequences throughout associated communities and ecosystems (Keith, Bailey, Lau, & Whitham, 2017; Shuster, Lonsdorf, Wimp, Bailey, & Whitham, 2006; Whitham et al., 2006, 1999, 2008). Knowing which genes and alleles drive these patterns is a major goal in the fields of community and ecosystem genetics (Whitham et al., 2008), and the genes regulating SPGs are prime candidates for realizing that goal. For example, phenolic glycosides are important defenses against a variety of foundation defoliators including gypsy moths and forest tent caterpillars (Hwang & Lindroth, 1997), but are beneficial to chrysomelid beetles (*Chrysomela confluens*) that sequester the plant's defenses for their own defense (Martinsen et al., 1998). The enzymatic steps linking leaf salicortin with beetle sequestered defensive chemistry have been described (Pasteels, Rowell‐Rahier, Braekman, & Dupont, 1991), and future discovery of *Populus* phenolic glycoside genes should reveal a metabolic pathway that originates in a foundation plant and continues in a foundation herbivore whose distribution and abundance can scale up to affect entire communities (Waltz & Whitham, 1997).

## 5 CONCLUSIONS

While our results are preliminary steps toward identifying SPG genes, our study has five important conclusions. First, it is the first study of salicinoid phenolic glycosides to use a full‐sib mapping population and a linkage map anchored to the *Populus* genome sequence to identify QTL interval for salicinoid phenolic glycosides. Second, it complements a recent association mapping study by Caseys et al. (2015) that used European *Populus* species to investigate the evolution of SPG diversity, and constraints therein, for a more distantly related section of the genus. Third, we identify a major likely Mendelian locus for HCH‐salicortin that provides a focal point for future research on SPG gene discovery. Fourth, our QTL intervals contain candidate genes known to be associated with compounds that are thought to play potential roles in the SPG pathway (i.e., hydroxycinnamoyl and its precursors or derivatives; Tsai, Harding, et al., 2006; Tsai, Kayal, et al., 2006) and are similar to those discussed by Chedgy et al. (2015). Finally, our results are an important first step toward linking genomic data with community‐level patterns—a major goal in the developing field of community genetics and evolution (e.g., Allen et al., 2012; Whitham et al., 2006, 2008). These conclusions emphasize the role of *Populus* as a model organism for studying the chemical ecology and evolution of foundation forest trees and for the advancement of the developing field of community genetics and genomics.

## CONFLICT OF INTEREST

None declared.

## AUTHOR CONTRIBUTIONS

TGW, PK, GDM, and SAW planned and designed the experiments; RLL and BJR conducted the chemical analyses and contributed to writing and editing; GDM and SAW conducted the experimental crosses and performed the QTL experiments; SPD and MSZ conducted genomic data analyses, and participated in writing and editing; GJA oversaw laboratory work and contributed to data analysis, writing, and editing; SAW wrote the manuscript.
