# Supplementary material for: Large effect quantitative trait loci for salicinoid phenolic glycosides in Populus: Implications for gene discovery
Source: Ecol Evol. 2018 Mar 8;8(7):3726–37. doi: 10.1002/ece3.3932 (PMC5901179; doi:10.1002/ece3.3932)
Supplement: Supplementary file 3 [file ECE3-8-3726-s003.docx]

**Supplemental Tables** can be downloaded from the Dryad Digital Repository: <https://doi.org/10.5061/dryad.8h67t>.

**Table S1. Candidate gene lists for all QTL**

Each worksheet in the file contains the gene lists for a particular interval. In cases where QTL intervals overlapped on the same chromosome, the +/- 1.3Mb interval pertains to the furthest/nearest point from the chromosome start (i.e. co-occurring peaks at 1Mb and 2Mb would have an interval ranging from 0Mb to 3.3Mb). Each table includes transcript name, gene name, gene start and end positions in bp, and general description. QTL peaks from our study, shared SSR makers from Caseys *et al.* (2015), and candidate genes are all shown in red text.

**Table S2. Proposed SPG candidate genes**

The table summarizes gene information for fourteen candidate genes that stand out with regard to their potential roles in SPG synthesis or regulation. Data are arranged by transcript name, gene name, gene start and end positions in bp, and a general description.
